# Oncolytic immunotherapy: multiple mechanisms of oncolytic peptides to confer anticancer immunity

**DOI:** 10.1136/jitc-2022-005065

**Published:** 2022-07-14

**Authors:** Tianyu Tang, Xing Huang, Gang Zhang, Tingbo Liang

**Affiliations:** 1 Zhejiang Provincial Key Laboratory of Pancreatic Disease, The First Affiliated Hospital, Zhejiang University School of Medicine, Hangzhou, Zhejiang, People's Republic of China; 2 Zhejiang Clinical Research Center of Hepatobiliary and Pancreatic Diseases, Hangzhou, People's Republic of China; 3 The Innovation Center for the Study of Pancreatic Diseases of Zhejiang Province, Hangzhou, People's Republic of China; 4 Department of Hepatobiliary and Pancreatic Surgery, The First Affiliated Hospital, Zhejiang University School of Medicine, Hangzhou, People's Republic of China; 5 Cancer Center, Zhejiang University, Hangzhou, People's Republic of China

**Keywords:** Immunotherapy, Tumor Microenvironment, Programmed Cell Death 1 Receptor, Oncolytic Virotherapy, Drug Therapy, Combination

## Abstract

Oncolytic peptides are highly effective on remodeling the tumor microenvironment and potentiating the anticancer immunity through multiple mechanisms, particularly by inducing immunogenic cell death. Intriguingly, a recent study demonstrates that LTX-315, one of the most promising and extensively studied oncolytic peptides, inhibits PD-L1 expression via ATP11B, thus enhancing the effectiveness of cancer immunotherapy by targeting the PD-1/PD-L1 axis. Therefore, this commentary discusses the broad effects and perspectives of oncolytic peptides on anticancer immunity, further highlighting the potential issues and directions of oncolytic peptides in cancer immunotherapy.

## Oncolytic immunotherapy

Physicians and radiologists noticed decades ago that local chemical or physical treatments such as radiotherapy can lead to the regression of distant and untreated malignant lesions.^1^ It was suggested that the destruction of local tumor cells has unique mechanisms and broad influence on the activation of body immune system that induces abscopal effects for the immune elimination of the tumor and long-term benefits in patients with cancer.^2^ Today, this concept has been supported by a wide range of studies, from preclinical models to clinical trials. Furthermore, local treatments have been constantly optimized for highly efficient and systemic immune-stimulatory effects. Among numerous strategies, the oncolytic immunotherapy emerges as one of the most promising approaches to boost antitumor immune response.

Viral and non-viral oncolysis have been widely investigated in cancer immunotherapy. Viral oncolysis, also known as oncolytic virus therapy, is considered as a major milestone in the development of immunotherapeutic approaches. Previous clinical trials showed that the local injection of oncolytic viruses induces a robust and systemic antitumor immune response, especially when combined with immune checkpoint inhibitors.^3^ In 2015, the Food and Drug Administration approved talimogene laherparepvec, a genetically modified virus encoding granulocyte-macrophage colony-stimulating factor, as the first oncolytic virus for the treatment of inoperable melanoma.^4^ However, the use of the oncolytic viruses is still limited because of multiple concerns, particularly in their undetermined safety, high cost and storage requirements, as well as complex administration procedures. In recent years, multiple forms of non-viral oncolysis have been reported and attracted increasing attention. The approaches include but are not limited to: (1) hyperthermic methods, causing coagulative necrosis in the central zone of tumors with a complete denaturation of the proteins (radiofrequency ablation, microwave ablation, laser therapy, and high-intensity focused ultrasonography); (2) cryotherapy, causing tumor cell death by freezing and thawing the tumor tissues with protein denaturation in a less extent; (3) irreversible electroporation, causing membrane rupture with a less change of antigen conformation by the delivery of high-voltage electrical pulses; (4) electrochemotherapy, causing both direct and indirect cellular injury by permeabilizing cells with electrodes following the application of chemotherapy; (5) photodynamic therapy, causing tumor cell apoptosis by inducing a high amount of reactive singlet oxygen using photosensitizer agents and high-intensity light; (6) local chemical agent injection, causing oncolytic cell death by cytotoxic chemical agents injected directly into the tumors (transarterial chemoembolization, radioembolization, and oncolytic peptides).^4^ These non-viral techniques not only cause distinct types of tissue destruction, inducing variable antitumor immunity compared with oncolytic virus therapies, but they are also relatively safe, inexpensive, and easily accessible.

## Antitumor immunity induced by oncolytic peptides

**Figure 1:**
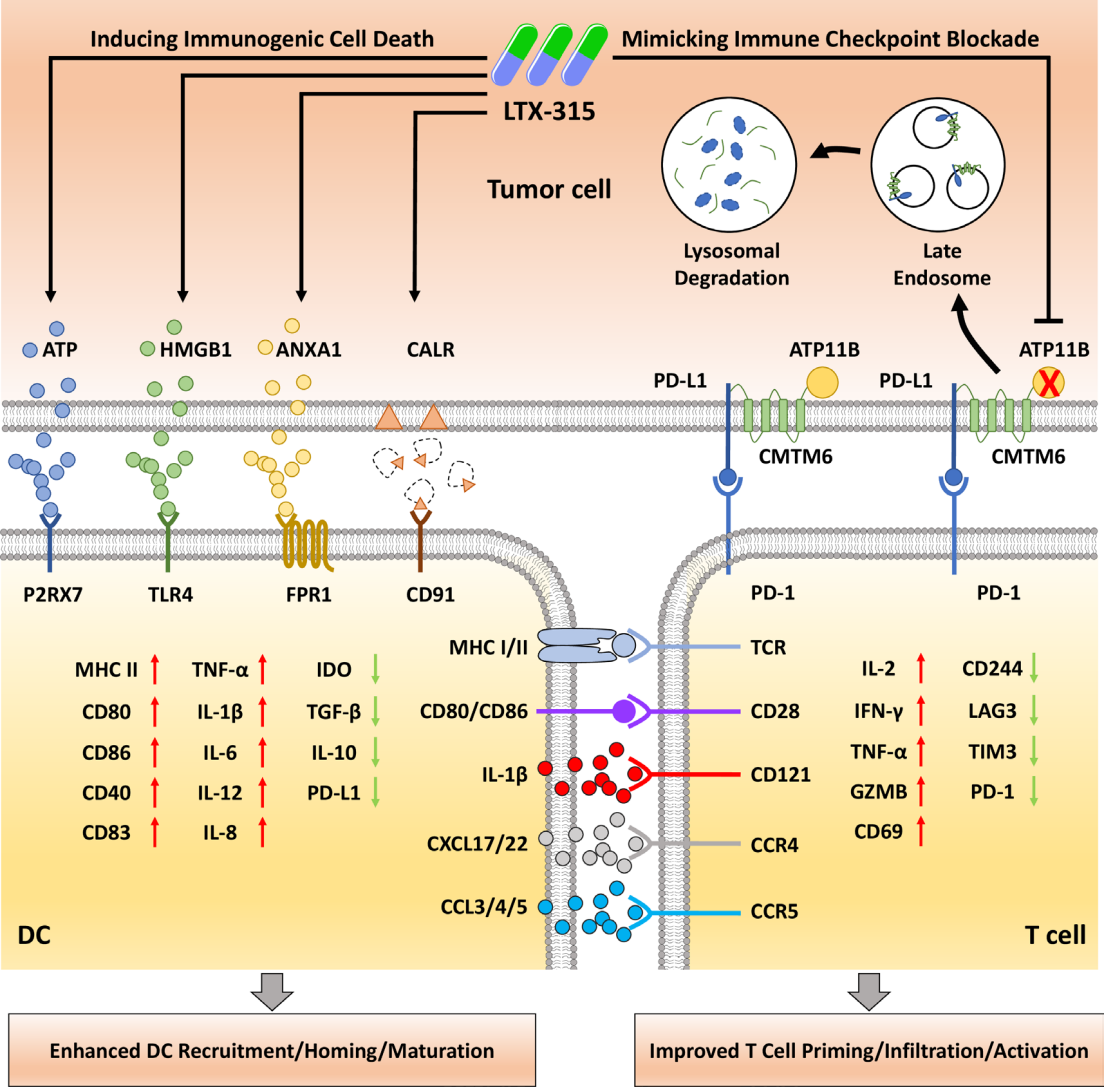
Multiple mechanisms of LTX-315 to confer anticancer immunity. LTX-315 treatment mimicks immune checkpoint blockade by inhibiting ATP11B/CMTM6 complex to promote PD-L1 lysosome-dependent degradation in tumor cells, meanwhile generating immunogenic oncolysis by inducing immunogenic cell death with the release of different types of DAMPs, including ATP, ANXA1, and HMGB1, as well as the exposure of CALR. These therapeutic responses cause both inhibition of negative immune regulation and promotion of positive immune regulation, and eventually cocontribute to the favorable immunobiological outcomes relevant to treatment success, including enhanced DC recruitment, homing, and maturation, as well as improved T cell priming, infiltration, and activation. ANXA1, annexin A1; APC, antigen-presenting cell; CALR, calreticulin; DAMP, danger-associated molecular pattern; DC, dendritic cell; GZMB, granzyme B; HMGB1, high mobility group B1; IDO, indoleamine 2,3-dioxygenase; LAG3, lymphocyte activation gene-3; MHC I/II, major histocompatibility complex I/II.

The oncolytic peptide is one of the most promising chemical oncolytic agents derived from natural antimicrobial peptides. These peptides include [D]-K3H3L9, LL37, Pardaxin, NK-2, AMP CSP32, RT53, LTX-315, LTX-401, DTT-205, and DTT-304, which are predominantly cytotoxic and can be injected directly into the targeted lesions.^5^ Despite the similar cytolytic effects, the oncolytic peptides are usually characterized by their own tropism for malignant cells due to their different affinity for various cellular membranes, including plasma membranes (RT53, LTX-315, HNP1-3, pardaxin, and AMP CSP32), mitochondrial membranes (LTX-315 and LL37), endoplasmic reticulum membranes (LTX-401 and Pardaxin,), Golgi membranes (LTX-401), and lysosome membranes (LTX-401, DTT-205, and DTT-304).^4 5^ Numerous pieces of evidence demonstrated that the oncolytic peptides exert anticancer activity by activating durable antitumor immunity and promoting immune infiltration in tumors through multiple mechanisms in different malignancies. Among the above promising oncolytic peptides, LTX-315 is the most extensively investigated agent in various malignancies due to its outstanding effect on promoting anticancer immunity and tumor growth inhibitions (Figure 1).

### Promotion of positive immune regulation

The local injection of oncolytic peptides is able to inflame the tumor microenvironment, partially by promoting positive immune regulation. Such effects on the activation of the immune system and the enhancement of the immune response are largely derived from the ability of the oncolytic peptides to induce immunogenic cell death (ICD).

ICD is characterized by the release of different types of danger-associated molecular patterns (DAMPs), including ATP, annexin A1, and high mobility group B1 (HMGB1), as well as the exposure of calreticulin.^6^ These signals operate on a series of receptors on antigen-presenting cells to promote antigen presentation, leading to the activation of tumor-targeted T cells. The ability of various oncolytic peptides to promote positive immune regulation through the induction of ICD has been investigated in multiple mouse tumor models. LTX-315 treatment induces ICD of melanoma tumor cells with an enhanced release of HMGB1.^7^ The injection of LTX-315 stimulates T-cell infiltration and results in complete regression of the tumors, and mice treated with LTX-315 are protected from tumor cell rechallenge compared with non-treated control animals. In addition, ELISA analysis of the mouse plasma revealed that LTX-315 treatment leads to an increased level of IL-6. Similar effects are also found using RT53, which is also reported to boost antitumor immunity by inducing ICD of melanoma cells.^8^ Moreover, the local administration of LTX-401 in hepatocellular carcinoma induces an increased release of DAMPs and enhances tumor-specific immune responses.^9^ LTX-401 treatment inhibits the growth of both local and distal metastatic lesions, indicating a robust abscopal antitumor effect. Furthermore, the injection of HNP1-3 in soft tissue sarcoma leads to ICD of tumor cells, increased immune infiltration in the tumors, and inhibition of tumor growth.^10^

In addition to the induction of ICD, the administration of such oncolytic peptides results in the differentiation and maturation of proinflammatory immune cells. The intratumoral injection of LTX-315 in breast tumor models leads to the reconfiguration of the tumor immune microenvironment and the inhibition of tumor growth in an NK-cell-dependent manner.^11^ Moreover, in vitro analyses showed that CSP32 regulates the polarization of murine macrophages and induces M1 macrophages with enhanced production of proinflammatory cytokines.^12^ Furthermore, the administration of LL37 triggers the activation and maturation of dendritic cells (DCs), by enhancing the transport of self-RNA released by dying cells to the endosomal compartments of DCs.^13^

### Inhibition of negative immune regulation

The local injection of oncolytic peptides also leads to the inhibition of the negative immune regulation at the molecular and cellular levels, hence reprograming the tumor immune microenvironment and inducing durable antitumor immunity.

Tumors upregulate a variety of immunosuppressive molecules to escape the immune surveillance, while the oncolytic peptide therapy stimulates the antitumor immune response by targeting these molecules. LTX-315 has recently been found to downregulate the expression of PD-L1, a representative immune-inhibitory checkpoint molecule, and enhance CD8^+^ T cell infiltration in pancreatic cancer.^14^ Interestingly, ATP11B has been identified as a potential target of LTX-315 for the regulation of PD-L1 expression. Briefly, in vitro analyses revealed that ATP11B interacts with PD-L1 in a CMTM6-dependent manner. ATP11B depletion induces the downregulation of PD-L1 through the lysosomal degradation mediated by CMTM6. Consistently, rescue assays demonstrated that LTX-315 downregulates PD-L1 expression by inhibiting the ATP11B/CMTM6/PD-L1 axis in pancreatic cancer. The in vivo models further confirmed the ability of LTX-315 to reactivate the tumor immune microenvironment and its synergic effect with PD-1/PD-L1 targeting therapy on tumor growth. Additionally, the injection of pardaxin in oral squamous cell carcinoma models induces a robust anticancer immunity by reducing the levels of the immunosuppressive PGE2, another representative immune-inhibitory checkpoint molecule. Pardaxin treatment synergizes with chemotherapy and significantly inhibits tumor growth in dimethyl benzanthracene-induced tumor model.^15^

In addition to the inhibition of immunosuppressive molecules, oncolytic peptides play a critical role in the depletion of immunosuppressive cells, including regulatory T cells (Tregs), suppressive myeloid cells, regulatory B cells, and myeloid-derived suppressor cells (MDSCs), so as to reshape the tumor microenvironment toward an immunoactive profile. According to a previous study, the intra-tumoral injection of LTX-315 significantly reduces the recruitment and accumulation of Tregs and MDSCs in mouse sarcoma models, although the infiltration of DCs does not significantly change. Such depletion of immune suppressive cells further facilitates the accumulation of polyfunctional T cells, sensitizing the tumor response to CTLA-4 blockade.^16^ Notably, the abundant reduction of Tregs results in a lower level of PD-1 expression in effector T cells, contributing to a better response to PD-1 blockade in LTX-315 treatment.

### Potential issues and directions of oncolytic peptides in clinical practice

Based on the promising therapeutic effects of LTX-315 in preclinical models, several exploratory clinical investigations have been conducted on distinct malignancies. Jebsen *et al* reported a 29-year-old woman with a desmoid tumor treated with LTX-315.^17^ The injection of LTX-315 under ultrasound guidance induced a significant shrinkage of the tumor, with an increased CD8^+^ T cell infiltration. Spicer *et al* reported the first phase I clinical trial using this oncolytic peptide, in which 39 patients diagnosed with refractory solid tumors were enrolled and received LTX-315 treatment.^18^ Stable diseases were achieved in nearly half of the patients and 82% experienced abscopal effect in at least one single lesion. However, according to the Immune-Related Response Criteria, no objective response was observed in patients treated with LTX-315, indicating the limited effects on clinical tumor inhibition.

Despite the encouraging results in the use of oncolytic peptides in preclinical models, the unsatisfying outcomes in the clinical application of oncolytic peptides may be due to the following reasons: (1) The mechanisms underlying the ability of oncolytic peptides to reprogram immune microenvironment are still not fully understood, particularly those independent from membrane-targeted lytic effects. For instance, the alterations in the cGAS-STING signaling pathway or dominant immune checkpoint molecules in tumor cells resistant to oncolytic peptide treatments need to be further addressed. (2) The heterogeneity of the patient populations, the complexity of tumor immune microenvironment, and the diversity of targets highlight the importance of potential biomarkers in governing the treatments with oncolytic peptides. The identification of biomarkers for antitumor immune responsiveness may help the development of an appropriate therapeutic regimen and eventually improve the overall efficacy of the oncolytic peptide treatment in clinical practice.^19^ (3) The intratumoral injection, currently the most common approach for oncolytic peptide delivery, limits the application of oncolytic peptides in tumors with deep locations, although ultrasound and CT may provide some guidance. New delivery approaches, such as intravenous delivery systems based on tumor-targeting nanocarrier, may represent a preferable route of administration in patients with tumors in deep locations.

Altogether, cancer immunotherapy based on oncolytic peptides is at a preliminary stage in clinical practice. Further studies focusing on mechanism demonstration for enhancing their efficacy, biomarker identification for optimizing their regimen, and approach improvement for expanding their application may jointly facilitate the translation of the use of oncolytic peptides to the clinical setting in the future.
